# Environmental exposure to polybrominated biphenyl (PBB) associates with an increased rate of biological aging

**DOI:** 10.18632/aging.102134

**Published:** 2019-08-02

**Authors:** Sarah W. Curtis, Dawayland O. Cobb, Varun Kilaru, Metrecia L. Terrell, M. Elizabeth Marder, Dana Boyd Barr, Carmen J. Marsit, Michele Marcus, Karen N. Conneely, Alicia K. Smith

**Affiliations:** 1 Genetics and Molecular Biology Program, Laney Graduate School, Emory University, Atlanta, GA 30322, USA; 2 Department of Gynecology and Obstetrics, School of Medicine, Emory University, Atlanta, GA 30322, USA; 3 Department of Epidemiology, Rollins School of Public Health, Emory University, Atlanta, GA 30322, USA; 4 Department of Environmental Health, Rollins School of Public Health, Emory University, Atlanta, GA 30322, USA; 5 Department of Pediatrics, School of Medicine, Emory University, Atlanta, GA 30322, USA; 6 Department of Human Genetics, School of Medicine, Emory University, Atlanta, GA 30322, USA; 7 Department of Psychiatry and Behavioral Sciences, School of Medicine, Emory University, Atlanta, GA 30322, USA

**Keywords:** endocrine-disrupting compound, EDC, age acceleration, DNA methylation age, sex-specific, brominated flame retardant

## Abstract

Advanced age increases risk for cancer, cardiovascular disease, and all-cause mortality. However, people do not age at the same rate, and biological age (frequently measured through DNA methylation) can be older than chronological age. Environmental factors have been associated with the rate of biological aging, but it is not known whether persistent endocrine-disrupting compounds (EDCs) like polybrominated biphenyl (PBB) would associate with age acceleration. Three different epigenetic age acceleration measures (intrinsic, extrinsic, and phenotypic) were calculated from existing epigenetic data in whole blood from a population highly exposed to PBB (N=658). Association between serum PBB concentration and these measures was tested, controlling for sex, lipid levels, and estimated cell type proportions. Higher PBB levels associated with increased age acceleration (intrinsic: β=0.24, 95%CI=0.01-0.46, p = 0.03; extrinsic: β=0.39, 95%CI=0.12-0.65, p = 0.004; and phenotypic: β=0.30, 95%CI=0.05-0.54, p = 0.01). Neither age when exposed to PBB nor sex statistically interacted with PBB to predict age acceleration, but, in stratified analyses, the association between PBB and age acceleration was only in people exposed before finishing puberty and in men. This suggests that EDCs can associate with the biological aging process, and further studies are warranted to investigate other environmental pollutants’ effect on aging.

## Introduction

Endocrine-disrupting compounds (EDCs) are a broad class of chemicals that are defined by their ability to disrupt the endocrine system by interfering with or mimicking endogenous hormone binding, synthesis, or transport [1]. Exposure to EDCs is common given that they are present in pesticide formulations, plastics, personal care products, flame retardant mixtures, and household dust [2,3]. Many studies have also shown that increased EDC exposure in human populations is associated with an increased incidence of hormone-related and developmental problems, especially in people exposed when they are younger [4–11]. Additionally, recent studies have demonstrated that increased exposure to EDCs is associated with an increased risk for age-related disorders such as cancer, Parkinson’s disease, stroke, and cardiovascular disease [12–16]. However, it can be difficult to assess how environmental factors influence the aging process and the risk for age-related diseases.

To overcome this difficulty, biomarkers of age have been developed. There is a well-established association between aging and DNA methylation [17–26]. Therefore, epigenetic clocks, developed from DNA methylation at specific sites across the genome, are becoming popular biomarkers of age [27]. Using these epigenetic clocks, methylation-based estimates of age can be predicted, and the difference between this predicted age and chronological age is defined as age acceleration. Several different measures of age acceleration have been constructed from DNA methylation data, including intrinsic age acceleration [28], extrinsic age acceleration [29], and phenotypic age acceleration [30]. Extrinsic age acceleration is based on 71 CpGs, which were selected from an elastic net regression of age in blood cells, and, therefore, is not well-suited as an estimate of age in non-blood tissues [29]. Intrinsic age acceleration is based on 353 CpGs, which were selected from an elastic net regression of age in multiple tissue types, making it more robust to age-related changes in blood composition and generalizable across most human tissues [28]. Phenotypic age acceleration uses 513 CpGs to capture information from ten age-related clinical characteristics (chronological age, albumin, creatinine, glucose, C-reactive protein levels, lymphocyte percentage, mean cell volume, red blood cell distribution width, alkaline phosphatase, and white blood cell count). Like the extrinsic measure, phenotypic age acceleration was also designed for use in blood samples [30].

All these epigenetic age acceleration measures capture different aspects of biological aging and have been associated with many age-related health outcomes. For example, an age acceleration greater than five years (by any of the three measures) has been associated with an 11-21% increase in all-cause mortality [30–32] and is associated with age-related diseases like Alzheimer’s disease, Huntington’s disease, and cardiovascular disease [30,33–35]. Additionally, increased age acceleration has been associated with increased cancer risk, including an increased risk for endocrine-related cancers like breast cancer [36–41]. Furthermore, positive age acceleration has been associated with advanced pubertal development (Tanner stages and age of menarche) [42,43], as well as with earlier menopause [44]. It is also higher in breast tissue compared to blood [45], suggesting that hormonal factors like estrogen could also be influencing the aging process. Together, this indicates that epigenetic age acceleration may be a biomarker for biological age and that alterations to epigenetic age acceleration can put people at risk for a wide variety of other health problems [46].

Because of the links between age acceleration and adverse health outcomes, research has been done to investigate what environmental factors can influence the age acceleration rate. For example, increased exposure to environmental pollutants like air pollutants is associated with increased age acceleration, and that association is moderated by sex [47–50]. However, even though age acceleration has been associated with EDC-linked hormone-dependent health outcomes like breast cancer and pubertal development [38,41,43], and EDC exposure is now being associated with age-related health conditions [12–16], there is a paucity of data on the effect of EDCs on age acceleration. The only study of EDCs and age acceleration reported that 2,2-bis(4-chlorophenyl)-1,1-dichloroethylene (p,p’-DDE) and transnonachlor (TNC), two organochlorine pesticides, were associated with higher age acceleration, but hexachlorobenzene (HCB) was not associated [51]. However, given that exposure to EDCs is common in the modern world [2,3], more research is needed to determine whether exposure to different types of EDCs are also linked to increased age acceleration, potentially increasing the risk for age-related health disorders.

To investigate the association between environmental exposure to EDCs and various measures of age acceleration, we utilized samples collected as part of the Michigan Polybrominated Biphenyl (PBB) Registry. The Michigan PBB Registry contains nearly 7,000 individuals who were highly exposed to PBB, an EDC, in the 1970s when it was accidentally added to farm animal feed and thus introduced into the food supply. Because of the long biological half-life of PBB (median of 13.5 years), a majority of the members of the Michigan PBB Registry still have PBB levels higher than 95% of the general US population [52–54]. In addition, because the timing of the contamination is well-documented, the age at exposure can be examined. Therefore, this registry offers a unique opportunity to analyze the association between age acceleration and EDC exposure. Previous research in this cohort has linked increased PBB exposure to earlier age of menarche, genitourinary conditions, thyroid dysfunction, and cancer [9–11,55–58]. PBB levels have also been associated with differences in DNA methylation proportions at sites enriched for estrogen signaling [59]. Additionally, many of these health outcomes are sex-specific, and people who were exposed when they were younger have been found to be more vulnerable than those exposed when they were older [9,11,60–62]. Age acceleration is associated with hormonal changes [41,43,63], and many of the health problems that PBB is associated with are also age-related, like pubertal development, lymphoma, and breast cancer [10,57,58,62]. However, a majority of the research conducted on PBB exposure has focused on reproductive and hormone-related health outcomes. Therefore, we tested whether PBB would associate with epigenetic age acceleration which could indicate that the hormone disruption associated with PBB may increase risk for other age-related health outcomes. Additionally, we tested if age at exposure or sex would interact with PBB exposure to predict increased age acceleration, similar to other health outcomes reported with PBB exposure.

## RESULTS

### Study population demographics

Participants of this study were highly exposed to PBB (range: 0.01-236.74 ng/mL, Table 1), compared to the general US population (median = 0.026 ng/mL). The 4 measured PBB congeners were positively correlated with each other (r = 0.23-0.99, p < 0.05). There were more female than male participants, and the average age at sample collection was 54 years (range: 18-88 years). Because a majority of the cohort was exposed to PBB during an accident in 1973, age when exposed to PBB is highly correlated with current age (r = 0.98, p < 2.2e-16). Older age at sample collection was correlated with higher PBB (r = 0.25, p < 2.2e-16), and men had higher levels of PBB compared to women (p = 4.54e-7). A majority of the cohort (97%) was of White/Non-Hispanic ancestry, which is representative of the population of rural Michigan in the 1970s.

**Table 1:** Characteristics of subset of Michigan PBB Registry with epigenetic data.

Study Population Demographics		
N		658
Number male^a^		277 (42.09%)
Current age (years)^b^		54.28 ± 12.74
Age when exposed to PBB (years) ^b^		15.18 ±11.55
Total PBB level (ng/mL or ppb)^c^		0.48 (4.71)
Total PBB level (ng/g lipids) ^c^		73.09 (4.95)
Intrinsic Age Acceleration (years)		-0.01 ± 4.55
Extrinsic Age Acceleration (years)		-0.04 ± 5.67
Phenotypic Age Acceleration (years)		-0.01 ± 5.05
Race/Ethnicity^a^		
	White/Non-Hispanic	638 (96.96%)
	White/Hispanic	20 (3.03%)

^a^Frequency and percentage

^b^Mean and standard deviation

^c^Geometric mean and geometric standard error

### Age acceleration measures

**Figure 1:**
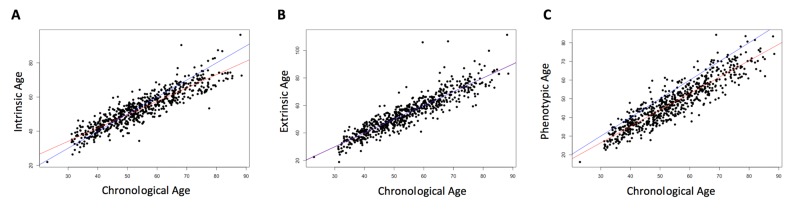
**Correlation of chronological age and biological age.** Chronological age was positively correlated with each of the three epigenetically-predicted ages. Intrinsic age and chronological age were highly correlated (r = 0.91, p < 2.2e-16, (**A**), as were extrinsic age and chronological age (r = 0.91, p < 2.2e-16, (**B**), and phenotypic age and chronological age (r = 0.91, p < 2.2e-16, (**C**). The red line indicates the regression of the epigenetically-predicted age on the chronological age. The blue line is a 1:1 line for comparison.

**Figure 2:**
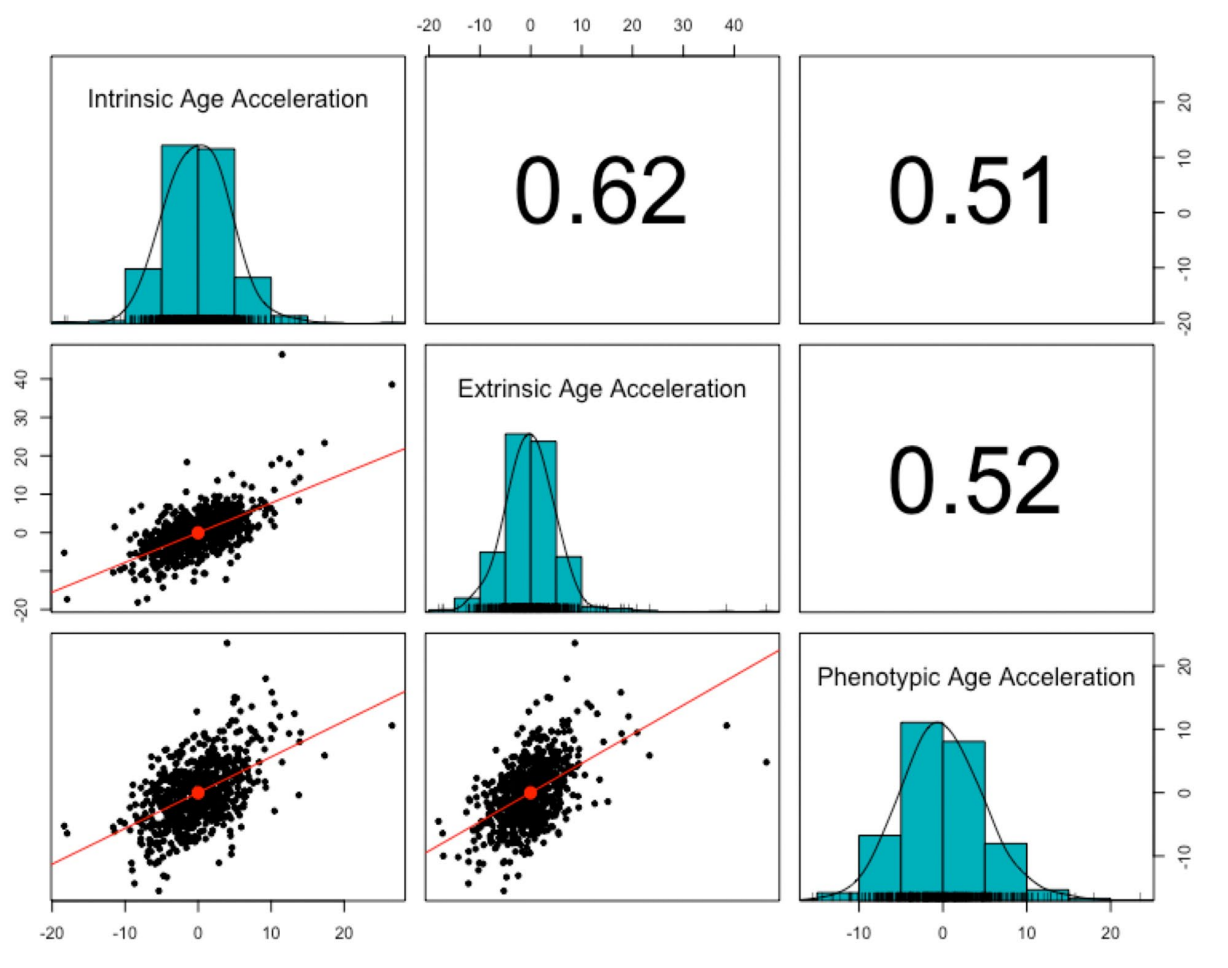
**Correlation of age acceleration measures.** The three epigenetic measures of age acceleration were all positively correlated with each other. Phenotypic age acceleration had the lowest correlation with the other two (r = 0.51, p <2.2e-16 with intrinsic age acceleration; r = 0.52, p <2.2e-16 with extrinsic age acceleration). Intrinsic age acceleration and extrinsic age acceleration had the highest correlation (r = 0.62, p < 2.2e-16).

Three measures of epigenetic age were calculated from the DNA methylation data: intrinsic, extrinsic, and phenotypic. All three were highly correlated with chronological age (r = 0.91, p < 2.2e-16, Figure 1). The three age acceleration measures (the residual from the regression of epigenetic age on chronological age) did not have significantly different means and standard deviations (p = 0.92-0.99, Table 1), and had a positive, moderate correlation with each other (r = 0.51-0.62, p < 2.2e-16, Figure 2). Many members of the cohort had an age acceleration measure that was greater than 5 years (intrinsic age acceleration: N = 78 (11.85%); extrinsic age acceleration: N = 90 (13.67%); phenotypic age acceleration: N = 97 (14.74%); all three measures: N = 22 (3.34%)). Intrinsic age acceleration was positively associated with being male and CD8T cell type proportions, and negatively associated with CD4T cell type proportions (Table S1). Extrinsic age acceleration was positively associated with being male, and B cell, NK cell, monocyte, and granulocyte proportions, and negatively associated with CD4T cell type proportions (Table S1). Phenotypic age acceleration was positively associated with monocyte and granulocyte proportions, and negatively associated with lipid levels and CD8T, CD4T, and B cell proportions (Table S1). These associations are largely consistent between the different measures, although the stronger association between extrinsic and phenotypic age acceleration and cell type proportions is most likely due to these measures reflecting age-related changes in immune cell composition.

### Age acceleration associates with current PBB level

**Figure 3:**
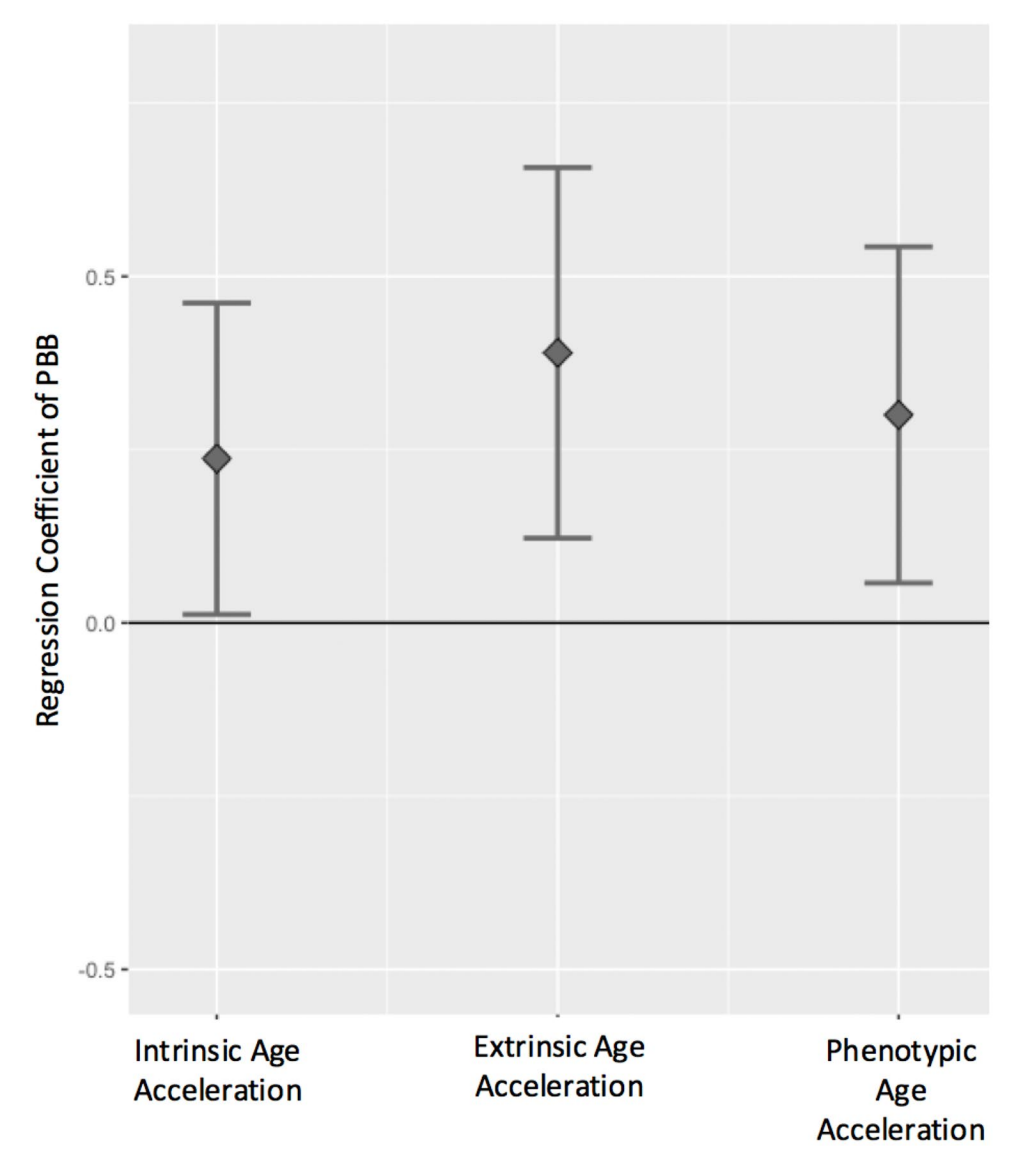
**Age acceleration positively associates with PBB exposure.** The regression coefficient and 95% confidence interval (y-axis) for PBB and each the age acceleration measures, controlling for sex, total lipid levels, and estimated cell types. PBB was positively associated with intrinsic age acceleration (t = 2.07, p = 0.03), extrinsic age acceleration (t = 2.86, p = 0.004), and phenotypic age acceleration (t = 2.43, p = 0.01).

All three measures of age acceleration were positively associated with total serum PBB levels, even after controlling for sex, cell type estimates, and lipid levels, such that high age acceleration was more common in people with higher levels of PBB. For every natural log unit increase in PBB exposure, there is a 0.24 year increase in intrinsic age acceleration (β=0.24, 95% CI=0.01-0.46, p = 0.03), a 0.39 year increase in extrinsic age acceleration (β=0.39, 95% CI=0.12-0.65, p = 0.004), and a 0.30 year increase in phenotypic age acceleration (β=0.30, 95%CI=0.05-0.54, p = 0.01; Figure 3; Figure S1; Table S2). These associations were consistent if PBB-153 (the congener detected in the majority of the participants) was analyzed with each age acceleration measure (Figure S2), if total lipid level was not included in the model, or if age was included as an additional covariate (Table S2).

### Age of exposure does not interact with PBB to predict age acceleration

Exposure to PBB primarily happened during a single contamination incident and previous research has shown that some of the effects of PBB vary based on age when exposed to PBB [11]. Therefore, we tested whether PBB exposure level and age when exposed to PBB interacted to predict age acceleration. No statistically significant interaction was found, but in stratified analyses the association between PBB and all three age acceleration measures was only significant in the subset that was exposed before finishing puberty (Table 2, Table S3).

**Table 2:** Results from the stratified analyses and the interaction model between age at exposure and PBB.

	Exposed to PBB before finishing puberty (N = 386)		Exposed to PBB after finishing puberty (N = 272)		Interaction	
	β (95% CI)	P-value	β (95% CI)	P-value	β (95% CI)	P-value
Intrinsic Age Acceleration	0.2916 (0.0214, 0.5617)	0.03	0.0668 (-0.3513, 0.4850)	0.75	-0.0085 (-0.0265, 0.0095)	0.35
Extrinsic Age Acceleration	0.2853 (0.0029, 0.5677)	0.04	0.4567 (-0.0845, 0.9979)	0.09	0.0084 (-0.0129, 0.0297)	0.43
Phenotypic Age Acceleration	0.2776 (-0.0084, 0.5638)	0.05	0.3631 (-0.0931, 0.8195)	0.11	0.0082 (-0.0111, 0.0276)	0.40

### Interactions with sex and PBB

Previous research in the PBB cohort has indicated that there may be sex-specific health effects with PBB exposure [10,56,62], and studies of other pollutants have found sex-specific associations with age acceleration [50], therefore, we tested whether PBB and sex interacted to predict any of the age acceleration measures. There were no statistically significant interactions between PBB and sex. However, in stratified analyses, the association between PBB and intrinsic age acceleration and extrinsic age acceleration was only significant in men (Table 3, Table S3).

**Table 3:** Results from the stratified analyses and the interaction model between sex and PBB.

	Male (N=277)		Female (N=381)		Interaction	
	β (95% CI)	P-value	β (95% CI)	P-value	β (95% CI)	P-value
Intrinsic Age Acceleration	0.4242 (0.0551, 0.7933)	0.02	0.0643 (-0.2184, 0.3471)	0.65	0.3742 (-0.0722, 0.8205)	0.10
Extrinsic Age Acceleration	0.6236 (0.1881, 1.0590)	0.005	0.1649 (-0.1683, 0.4982)	0.33	0.4786 (-0.0521, 1.009)	0.07
Phenotypic Age Acceleration	0.3035 (-0.0932, 0.7003)	0.13	0.2801 (-0.0297, 0.5901)	0.07	0.0639 (-0.4187, 0.5466)	0.40

## DISCUSSION

This is the first study to report that PBB is associated with the rate of biological aging. This study utilized samples collected as part of the Michigan PBB Registry, a cohort of people who have PBB levels well above the national average due to exposure during an agricultural accident 40 years ago. Increased current levels of PBB associated with increased intrinsic, extrinsic, and phenotypic age acceleration, even when controlling for sex, lipid levels, and cell type proportions. These associations were consistent if just PBB-153 was analyzed, when serum lipids were not included as a covariate or when age was included as a covariate, supporting the robustness of these results. Finding that increased PBB exposure associates with increased age acceleration is consistent with previous studies in the Michigan PBB Registry that show an association between PBB and DNA methylation proportion [59,64], as well as most studies between age acceleration and environmental pollutants, like air pollution [47–50], and organochlorine pesticides [51]. It is also consistent with epidemiological studies that have found associations between PBB and cancer and pubertal development, both of which have been previously reported to associate with age acceleration [10,32,37,41,43,57,58,62,63]. This study further adds to the literature on EDC exposure and age acceleration by also analyzing phenotypic age acceleration, since the previous study only analyzed intrinsic and extrinsic age acceleration. Finding a positive association between PBB and all three epigenetic biomarkers demonstrates that PBB can not only impact epigenetic marks but may also influence the rate of biological aging.

The association between PBB and increased age acceleration could be caused by many biological mechanisms that cannot be directly tested in this study. Previous research in the population has indicated that PBB could be weakly estrogenic [10,57,59], and DNA methylation age is higher in tissues with higher estrogen levels like breast tissue [45]. Therefore, it is possible that the hormone dysregulation associated with PBB exposure could be the mechanism by which PBB associates with an increased rate of biological aging. Additionally, previous research has shown that PBB is related to immune dysfunction [58,59,65]. Age acceleration is also associated with immune function. Previous studies have reported a positive association between age acceleration and inflammatory markers, extrinsic and phenotypic age acceleration reflect age-related changes in immune cell composition, and all three measures associated with immune cell type proportions in this study [27,30,66]. Therefore, it is also possible that the immune dysfunction associated with PBB could lead to increased age acceleration. This could also explain why extrinsic and phenotypic age have a stronger association with PBB than intrinsic age acceleration does since they reflect more changes in immune function and cell type proportions [27]. However, neither of these mechanisms can be directly tested in study with human participants, and given the role of estrogen has in regulating the immune system [67–72], it is probable that PBB’s effect on both estrogen signaling and the immune system could explain the association between PBB and biological aging.

Finding a positive association between PBB and age acceleration measures is concerning given that age acceleration is associated with many adverse health outcomes, some of which have already been associated with PBB exposure. For example, both age acceleration and PBB exposure are associated with early age of menarche, which increases the risk for all-cause mortality, cardiovascular disease, and cancer [10,43,73–75]. Additionally, both are associated with increased cancer rates, particularly breast cancer [36–40,57,58]. This supports that in addition to hormone-related health problems, PBB may also associate with age-related health problems. It is important to note that increased age acceleration is also associated with other age-related health problems, such as Alzheimer’s disease [33], Huntington’s disease [34], cardiovascular disease [35], and all-cause mortality [31,32]. These conditions have not been studied in connection to PBB exposure, however, it is possible that because PBB exposure is associated with increased age acceleration, individuals exposed to PBB may have an increased risk for these conditions as well. However, these other health conditions and their association with PBB have not been directly measured or tested in this population, and therefore more research is needed to test whether PBB exposure associates with increased risk for aging-related diseases.

Because PBB exposure happened during a unique contamination incident, we were able to estimate the age each of the participants were when they were first exposed to PBB. Many of the adverse health outcomes that are associated with PBB have been found in the people who were exposed younger [10,11,61,62,76,77], and if the study population was stratified by age when exposed, the association between PBB and intrinsic and extrinsic age acceleration measures was only significant in the people who were exposed at younger ages, consistent with previous studies. However, there was no significant statistical interaction between age when exposed and PBB level. Therefore, there is no evidence that the association between PBB and age acceleration is moderated by age of exposure to PBB. While this may be an issue with statistical power for testing interactions, it is more likely that age acceleration is associated with PBB regardless of when exposure occurs, and that whatever mediates the association between age acceleration and PBB affects people regardless of their age when first exposed to PBB.

We also found that if we stratified our population by sex, the associations between PBB and intrinsic and extrinsic age acceleration were only significant in men. If hormonal dysregulation from PBB exposure is what mediates the association with increased age acceleration, one would expect there to be sex-specific associations with PBB and age acceleration. Additionally, many weakly estrogenic compounds do have stronger effects in males, although the research is inconsistent [78,79]. However, none of the interaction terms were statistically significant. This is contrary to a previous report of air pollution and age acceleration, which found interactions with sex [50]. That study, though, had almost three times the sample size of this study. Therefore, given that the previous study was better powered, and the interactions terms with intrinsic and extrinsic age acceleration in this study were borderline significant, it is possible that there would be a significant interaction between PBB and sex in a larger study population, with men being more susceptible to higher age acceleration from PBB exposure. It is also possible that hormonal dysregulation does not influence the association between PBB and aging or that the exposure to PBB is so high in this population that both sexes are similarly affected. More studies with larger sample sizes are needed to test whether there is a significant interaction with sex.

This study does have several limitations. First, DNA methylation was only measured from whole blood samples, and therefore it is not known if PBB exposure would have a different impact on the aging of different tissues. Additionally, our sample size may have been too small to adequately test for interactions with sex or age of exposure. Furthermore, because age-related health concerns have only recently been associated with EDC exposure, information on many age-related conditions, like cardiovascular disease and cognitive decline, have not been collected from participants. Many participants also lacked information on potential confounders like smoking status and weight, and we were thus unable to control for these variables in our models. Future studies with this cohort, and other cohorts exposed to EDCs, should consider collecting broad information on health conditions.

In conclusion, we were able to find that PBB levels are positively associated with intrinsic, extrinsic, and phenotypic age acceleration. While the biological mechanism behind this association remain unknown, immune dysfunction and hormone dysregulation may contribute. Unlike other reports of age acceleration and environmental pollutants and previous studies in the Michigan PBB Registry, we did not find any significant interactions between age when exposed or sex and PBB. Because alterations in age acceleration have been associated with numerous health conditions, including Alzheimer’s disease [33], cancers [36–40], and earlier age of puberty [43], this could indicate that high exposure to these endocrine-disrupting compounds could increase people’s risk for developing these health problems. More research on the prevalence of age-related health conditions and mortality in the Michigan PBB Registry is warranted. Furthermore, studies of other environmental pollutants should also be conducted to test whether they also impact the rate of biological aging.

## MATERIALS AND METHODS

### Participant selection

As previously described [59], participants were selected from the Michigan PBB Registry, which was started by the Michigan State Health Department (now the MDHHS) after the PBB contamination was discovered. The MDHHS recruited individuals that either lived on farms quarantined because of PBB contamination or obtained food from quarantined farms, or were chemical workers or family members of chemical workers. This registry, now at Emory University, has continued to enroll participants, collect biological samples, and survey health outcomes (http://pbbregistry.emory.edu/). For the current analyses, participants were selected from the registry if they met the following criteria: 1) they were exposed to PBB prior to being 50 years old, 2) they had a recent (2004-2015) buffy coat or whole blood sample available for DNA extraction, and 3) they had current PBB and lipid level measurements for their serum. A total of 666 participants met these criteria and were selected for this study. PBB measurement and DNA extraction (as described below) were conducted on these samples at the same time and not as samples were collected.

### Exposure assessment

209 possible congeners of PBB exist and are defined based on the number and position of the bromine molecules around the biphenyl rings [80]. In the technical mixture of PBBs that was added to the food supply in Michigan, the primary congener was PBB-153 [80–82]. Exposure to four congeners of PBB (PBB-153, PBB-101, PBB-77, and PBB-180) was previously assessed in members of this registry using gas chromatography-tandem mass spectrometry [83]. The limit of detection (LOD) was 2 pg/mL for PBB-153, 4.5 pg/mL for PBB-77, 3.9 pg/mL for PBB-101, and 5.6 pg/mL for PBB-180. The extraction recovery ranged from 83.2-99.2%. The accuracy ranged from 89-119% and the precision ranged from 2.8-8.5%.

For the purposes of this study, the value for congeners below the LOD in a sample was imputed as the LOD divided by the square root of 2 (PBB-153: N = 8; PBB-101: N = 70; PBB-77: N = 63; PBB-180: N = 655) [84]. The congeners were then summed to give a total PBB value per person. Because the distribution of PBB serum levels was skewed, the natural log of the serum levels was used in analyses. Because one congener, PBB-153, makes up the majority of the PBB mixture and was detected in the majority of participants, we also conducted sensitivity analyses with PBB-153 levels alone.

### Lipid measurement

A Triglyceride Quantification Assay Kit (Abnova Corporation) was used to measure the total triglyceride content in serum, and a Cholesterol Assay Kit (Caymen Chemical Company) was used to measure total cholesterol content in serum. Both were done according to manufacturer’s instructions. Total lipid amount was calculated based on these components as described elsewhere [85].

### MethylationEPIC array

As previously described [59], peripheral blood samples were collected from participants as part of the ongoing Michigan PBB Registry between 2004-2015. Blood was spun at 3,000 rpm to separate the plasma from the buffy coat. Buffy coats were aliquoted and stored at -80°C. DNA was extracted from buffy coat samples using the QIAamp DNA Blood Mini Kit (QIAGEN, Hilden, Germany). DNA was extracted from the buffy coats derived from the same blood samples as the serum used for the PBB measurements in order to limit confounding.

Methylation levels were measured from these DNA samples at >850,000 sites using the Infinium MethylationEPIC BeadChip (Illumina, San Diego, CA) [86]. Briefly, 1μg of DNA from participants’ buffy coats was bisulfite converted, amplified, fragmented, and hybridized to the BeadChip array according to the manufacturer’s instructions. DNA from a stable lymphoblast line was used as a technical replicate. For each individual sample at each probe, the methylation proportion (β) at that site was calculated from the methylated (M) and unmethylated (U) signal as β = M/(U+M). Six samples that were mismatches for sex, genetically identical to other samples, or flagged for sample contamination were removed. The 57 SNP probes were removed from the dataset. This resulted in a final dataset of 658 participants and 816,999 probes. Cell type estimation was calculated for each sample using Houseman’s method by first using BMIQ to adjust for probe type and ComBAT to adjust for batch effects [87,88], and then estimating cell type proportion with methylation signals from CpGs that are distinct in blood cell types [28,89]. The DNA methylation data can be accessed on NCBI’s Gene Expression Omnibus (GSE116339).

### DNA methylation age

Three measures of DNA methylation age were calculated. Intrinsic age was calculated as the linear combination of the beta values of 353 CpGs that have been previously shown to predict chronological age in multiple tissues by Horvath, *et al.* [28], times their effect size from the regression with chronological age. Extrinsic age was calculated similarly, but from 71 CpGs that have previously shown to predict chronological age in whole blood by Hannum, *et. al*. [29]. Phenotypic age (PhenoAge) was calculated similarly, but from 513 CpGs previously shown by Levine, *et. al.*, to predict a measure of phenotypic age built from age, immune function measures, and metabolic measures [30]. Phenotypic age acceleration was developed by Levine, *et. al.* by first developing a phenotypic age measure based on ten age-related clinical characteristics (chronological age, albumin, creatinine, glucose, C-reactive protein levels, lymphocyte percentage, mean cell volume, red blood cell distribution width, alkaline phosphatase, and white blood cell count), and then using elastic net to select 513 CpGs that predict this phenotypic age. Like the extrinsic measure, phenotypic age acceleration was also designed for use in blood samples, and so both of these measures may not be well-suited for other tissues. Intrinsic age acceleration, because it was developed in multiple tissues, is independent of age-related changes in blood cell composition. For this study, intrinsic and extrinsic age were calculated from the background-corrected beta values using Horvath’s publicly available online calculator (https://dnamage.genetics.ucla.edu). PhenoAge was calculated from the published coefficients for these 513 CpGs [30]. All three measure first calculate a DNA methylation based age, and then calculate the age acceleration from the residuals of the regression of DNA methylation age on chronological age.

### Statistical analysis

Linear regression models were used to test for association between PBB serum level (as the independent variable) and each of the three epigenetic age (as the dependent variable) in the 658 participants. Sex, lipid levels, and estimated cell type proportions were included as covariates [90]. Age was not used as a covariate since the dependent variable age acceleration, calculated from the residual, is independent of age. However, age was added to the models in sensitivity analyses. The association between PBB-153 and each age acceleration measure was also tested using the same covariates as above as a sensitivity analysis.

Because age when exposed to PBB can be estimated as the age of the participant in 1973, interaction between PBB and age of exposure was evaluated by the inclusion of an interaction term (age at exposure × total PBB level) to the model with PBB level, age when exposed, sex, total lipid level, and cell type estimates. The study population was then stratified into participants who were exposed before finishing puberty (age of exposure less than or equal to 16) and those who were exposed after finishing puberty (age of exposure greater than 16), consistent with previous studies in this cohort [53,54,91,92] and with the average age of ending puberty [93]. The association between current PBB level and age acceleration was then tested in each subset separately, with the same covariates as before. Additionally, interaction between exposure level and sex was evaluated by the inclusion of an interaction term (sex × total PBB level) to the model with exposure level, sex, total lipid level, and cell type estimates. The study population was then stratified by sex. The association between current exposure level and age acceleration was then tested in each subset separately, with the same covariates as before.

An alpha level of 0.05 was used to determine statistical significance. Correction for multiple tests was not done because age acceleration measures were correlated and thus did not constitute independent tests. All statistical models were run with R (3.6.0).

## Supplementary Material

Supplementary Figures

Supplementary Tables
