# Supplementary material for: Environmental exposure to polybrominated biphenyl (PBB) associates with an increased rate of biological aging
Source: Aging (Albany NY). 2019 Aug 2;11(15):5498–517. doi: 10.18632/aging.102134 (PMC6710070; doi:10.18632/aging.102134)
Supplement: Supplementary Figures [file aging-11-102134-s002.pdf]

SUPPLEMENTARY FIGURES

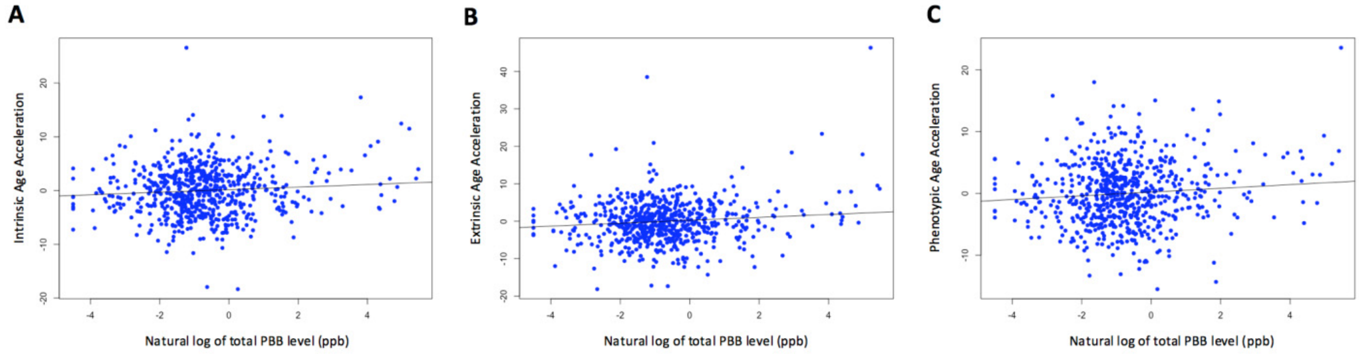

Figure S1. Regression of each age acceleration measure and total PBB. The association between each age acceleration measure and total PBB was tested, controlling for sex, total lipid levels, and cell types. Between total PBB and intrinsic age acceleration there was a positive, significant association (t = 2.07, p = 0.03, **A**). Between total PBB and extrinsic age acceleration there was a positive, significant association (t = 2.86, p = 0.004, **B**). Between total PBB and phenotypic age acceleration there was a positive,

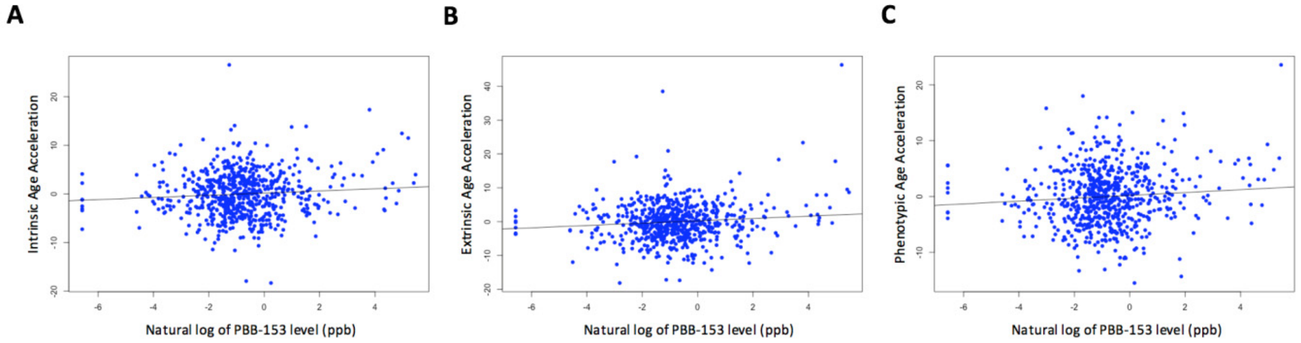

Figure S2. Regression of each age acceleration measure and PBB-153. The association between each age acceleration measure and PBB-153 (the congener that a majority of participants were most exposed to) was tested, controlling for sex, total lipid levels, and cell types. Between PBB-153 and intrinsic age acceleration there was a positive, significant association (t = 2.11, p = 0.03, **A**). Between PBB-153 and extrinsic age acceleration there was a positive, significant association (t = 2.74, p = 0.006, **B**). Between PBB-153 and phenotypic age acceleration there was a positive, significant association (t = 2.23, p = 0.02, **C**).
