# Supplementary material for: Environmental exposure to polybrominated biphenyl (PBB) associates with an increased rate of biological aging
Source: Aging (Albany NY). 2019 Aug 2;11(15):5498–517. doi: 10.18632/aging.102134 (PMC6710070; doi:10.18632/aging.102134)
Supplement: Supplementary Tables [file aging-11-102134-s001.pdf]

SUPPLEMENTARY TABLES

Table S1. Correlation of age acceleration measures and potential covariates.

Covariate	Intrinsic Age Acceleration		Extrinsic Age Acceleration		Phenotypic Age Acceleration	
	T-statistic	P-value	T-statistic	P-value	T-statistic	P-value
Current Age	0.12	0.90	0.31	0.75	-0.08	0.92
Sex	1.97	**0.04**	3.86	**1.20e-4**	1.45	0.14
CD8T	4.14	**3.81e-05**	0.22	0.82	-3.08	**2.15e-3**
CD4T	-4.17	**3.45e-05**	-8.30	**5.68e-16**	-8.50	**<2.2e-16**
B cells	0.24	0.80	4.42	**1.11e-5**	-2.43	**0.01**
NK	0.87	0.38	2.88	**4.08e-3**	-0.04	0.96
Monocytes	1.17	0.23	4.46	**9.62e-6**	3.34	**8.65e-4**
Granulocytes	1.25	0.21	3.01	**2.71e-3**	6.31	**5.08e-10**
Total lipid levels	-1.94	0.05	-1.37	0.17	-2.99	**2.81e-3**

Table S2. Association of environmental chemical exposure and age acceleration measures.

		Intrinsic Age Acceleration		Extrinsic Age Acceleration		Phenotypic Age Acceleration	
Model	Variables	β (95% CI)	P	β (95% CI)	P	β (95% CI)	P
1	Total PBB (ppb)	0.2370 (0.0122, 0.4617)	0.03	0.3897 (0.1223, 0.6570)	0.004	0.3001 (0.0575, 0.5426)	0.01
	Sex	0.5086 (-0.2312, 1.2484)	0.17	0.6730 (-0.2069, 1.5530)	0.13	-0.6495 (-1.4478, 0.1487)	0.11
	CD8T	25.2600 (16.4099, 34.1063)	3.07e-08	14.8372 (4.3128, 25.3615)	0.005	-6.4630 (-16.010, 3.0848)	0.18
	CD4T	-13.8500 (-20.0331, -7.6612)	1.29e-05	-28.0921 (-35.4499, -20.7343)	2.15e-13	-24.4400 (-31.1189, -17.7688)	1.78e-12
	B cell	2.4740 (-6.4084, 11.3556)	0.58	26.8977 (16.3330, 37.4624)	7.41e-07	-8.5650 (-18.1497, 1.018)	0.07
	NK	-0.6368 (-9.4042, 8.1307)	0.88	3.5203 (-6.9079, 13.9487)	0.50	-4.6880 (-14.1484, 4.7728)	0.33
	Mono	-0.7510 (-12.8930, 11.3910)	0.90	2.3074 (-12.1346, 16.7496)	0.75	0.9646 (-12.1373, 14.0665)	0.88
	Lipids (mg/dL)	-0.0014 (-0.0030, 0.0002)	0.09	-0.0010 (-0.0030, 0.0009)	0.29	-0.0026 (-0.0044, -0.0008)	0.004
2	Total PBB (ppb)	0.2472 (0.0224, 0.4719)	0.03	0.3974 (0.1304, 0.6643)	0.003	0.3190 (0.0754, 0.5624)	0.01
	Sex	0.5733 (-0.1635, 1.3101)	0.12	0.7217 (-0.1536, 1.5970)	0.10	-0.5297 (-1.3281, 0.2686)	0.19
	CD8T	25.4148 (16.5566, 34.2730)	2.63e-08	14.9550 (4.4321, 25.4778)	0.005	-6.1728 (-15.7705, 3.4250)	0.20
	CD4T	-13.6802 (-19.8714, -7.4890)	1.66e-05	-27.9666 (-35.3212, -20.6119)	2.65e-13	-24.1347 (-30.8428, -17.4265)	4.15e-12
	B cell	2.0482 (-6.8316, 10.9279)	0.65	26.5778 (16.0292, 37.1263)	9.59e-07	-9.3534 (-18.9746, 0.2678)	0.05
	NK	-0.6447 (-9.4239, 8.1346)	0.88	3.5144 (-6.9147, 13.9435)	0.50	-4.7025 (-14.2148, 4.8099)	0.33
	Mono	-0.1765 (-12.3158, 11.9629)	0.97	2.7396 (-11.6810, 17.1602)	0.70	2.0287 (-11.1243, 15.1816)	0.76
3	Total PBB (ppb)	0.2486 (0.0196, 0.4774)	0.03	0.4580 (0.1871, 0.7288)	0.0009	0.3701 (0.1247, 0.6154)	0.003
	Sex	0.5390 (-0.2097, 1.2877)	0.15	0.8529 (-0.0330, 1.7388)	0.05	-0.4652 (-1.2677, 0.3373)	0.25
	CD8T	25.8500 (15.8627, 33.8293)	7.93e-08	12.4000 (1.7706, 23.0303)	0.02	-8.9610 (-18.5897, 0.6680)	0.06

3	Total PBB (ppb)	0.2486 (0.0196, 0.4774)	0.03	0.4580 (0.1871, 0.7288)	0.0009	0.3701 (0.1247, 0.6154)	0.003
	Sex	0.5390 (-0.2097, 1.2877)	0.15	0.8529 (-0.0330, 1.7388)	0.05	-0.4652 (-1.2677, 0.3373)	0.25
	CD8T	25.8500 (15.8627, 33.8293)	7.93e-08	12.4000 (1.7706, 23.0303)	0.02	-8.9610 (-18.5897, 0.6680)	0.06
	CD4T	-13.9600 (-20.1658, -7.7579)	1.16e-05	-28.7700 (-36.1113, -21.4293)	5.26e-14	-25.1400 (-31.7888, -18.4893)	3.60e-13
	B cell	2.1550 (-6.8094, 11.1198)	0.63	25.0200 (14.4075, 35.6230)	4.41e-06	-10.5000 (-20.1041, -0.8863)	0.03
	NK	-0.0422 (-9.0859, 9.0013)	0.99	7.0360 (-3.6656, 17.7367)	0.19	-1.0840 (-10.7777, 8.6093)	0.82
	Mono	-0.3323 (-12.5793, 11.9147)	0.95	4.7830 (-9.7086, 19.2747)	0.51	3.5020 (-9.6246, 16.6296)	0.60
	Lipids (mg/dL)	-0.0013 (-0.0030, 0.0002)	0.10	-0.0009 (-0.0028, 0.0010)	0.36	-0.0024 (-0.0042, -0.0006)	0.007
	Age	-0.0082 (0.0389, 0.0223)	0.59	-0.0490 (-0.0852, -0.0127)	0.008	-0.0502 (-0.0830, -0.0173)	0.002
4	Total PBB (ppb)	0.3935 (0.0560, 0.7309)	0.02	0.3861 (-0.0129, 0.7853)	0.05	0.2962 (-0.0654, 0.6577)	0.10
	Sex	0.5968 (-0.1510, 1.3447)	0.11	0.7532 (-0.1313, 1.6378)	0.09	-0.5714 (-1.3728, 0.2301)	0.16
	CD8T	24.6500 (15.6840, 33.6124)	9.41e-08	12.0981 (1.4955, 22.7007)	0.02	-9.1390 (-18.7458, 0.4678)	0.06
	CD4T	-14.3500 (-20.5600, -8.1360)	6.85e-06	-28.870065 (-36.2174, -21.5227)	4.57e-14	-25.2000 (-31.8602, -18.5456)	3.36e-13
	B cell	1.6890 (-7.2790, 10.6568)	0.71	24.4920 (13.8849, 35.0990)	6.89e-06	-10.9200 (-20.5260, -1.3043)	0.02
	NK	1.2600 (-7.8058, 10.3249)	0.78	7.0372 (-3.6850, 17.7595)	0.19	-1.2550 (-10.9703, 8.4602)	0.79
	Mono	0.4604 (-11.7826, 12.7034)	0.94	5.148932 (-9.3317, 19.6296)	0.48	3.7390 (-9.3816, 16.8597)	0.57
	Lipids (mg/dL)	-0.0014 (-0.0031, 0.0002)	0.08	-0.0010 (-0.0030, 0.0008)	0.28	-0.0026 (-0.0044, -0.0008)	0.003
	Age exposed	-0.0285 (-0.0643, 0.0071)	0.11	-0.0574 (-0.0997, -0.0151)	0.007	-0.0561 (-0.0943, -0.0178)	0.004
	Age exposed × Total PBB	-0.0085 (-0.0265, 0.0095)	0.35	0.0084 (-0.0129, 0.0297)	0.43	0.0082 (-0.0111, 0.0276)	0.40
5	Total PBB (ppb)	0.0563 (-0.2548, 0.3675)	0.72	0.1585 (-0.2114, 0.5286)	0.40	0.2692 (-0.0672, 0.6056)	0.11
	Sex	0.7821 (-0.0256, 1.5898)	0.05	1.0229 (0.0625, 1.9833)	0.03	-0.6028 (-1.4761, 0.2705)	0.17
	CD8T	25.5300 (16.6890, 34.3742)	2.16e-08	15.1871 (4.6728, 25.7013)	0.004	-6.4160 (-15.9772, 3.1450)	0.18
	CD4T	-13.4500 (-19.6468, -7.2549)	2.32e-05	-27.5851 (-34.9524, -20.2179)	5.89e-13	-24.3800 (-31.0755, -17.6767)	2.43e-12
	B cell	2.0480 (-6.8373, 10.9326)	0.65	26.3528 (15.7881, 36.9174)	1.22e-06	-8.6380 (-18.2452, 0.9686)	0.07
	NK	-1.0250 (-9.7937, 7.7427)	0.81	3.0230 (-7.4027, 13.4489)	0.56	-4.7540 (-14.2349, 4.7265)	0.32
	Mono	-0.6533 (-12.7799, 11.4733)	0.91	2.4324 (-11.9867, 16.8515)	0.74	0.9813 (-12.1307, 14.0933)	0.88
	Lipids (mg/dL)	-0.0013 (-0.0030, 0.0002)	0.10	-0.0010 (-0.0029, 0.0009)	0.31	-0.0026 (-0.0044, -0.0008)	0.004
	Sex × Total PBB	0.3742 (-0.0722, 0.8205)	0.10	0.4786 (-0.0521, 1.0095)	0.07	0.0639 (-0.4187, 0.5466)	0.79

Table S3. Association of PBB and age acceleration measures in subsets.

		Intrinsic Age Acceleration		Extrinsic Age Acceleration		Phenotypic Age Acceleration	
Model	Variables	β (95% CI)	P	β (95% CI)	P	β (95% CI)	P
Exposed before puberty (N = 386)	Total PBB (ppb)	0.2916 (0.0215, 0.5617)	0.03	0.2853 (0.0029, 0.5677)	0.04	0.2777 (-0.0085, 0.5639)	0.05
	Sex	0.0451 (-0.8775, 0.9677)	0.92	0.3558 (-0.6088, 1.3203)	0.46	-1.0830 (-2.0604, -0.1056)	0.03
	CD8T	21.6902 (10.7857, 32.5947)	0.0001	6.6713 (-4.7288, 18.0714)	0.25	-8.0482 (-19.6001, 3.5038)	0.17
	CD4T	-13.6054 (-21.3471, -5.8637)	0.0006	-26.5916 (-34.6851, -18.4980)	3.23e-10	-26.4669 (-34.6682, -18.2655)	6.36e-10
	B cell	-3.0516 (-16.4872, 10.3839)	0.65	0.7104 (-13.3358, 14.7566)	0.92	-5.2824 (-19.5156, 8.9509)	0.46
	NK	-3.2475 (-14.5773, 8.0824)	0.57	9.3462 (-2.4986, 21.1909)	0.12	-8.3059 (-20.3084, 3.6967)	0.17
	Mono	6.0038 (-11.0282, 23.0357)	0.48	-0.8964 (-18.7025, 16.9096)	0.92	-4.6914 (-22.7346, 13.3518)	0.60
	Lipids (mg/dL)	-0.0008 (-0.0028, 0.0012)	0.42	-0.0009 (-0.0030, 0.0011)	0.37	-0.0019 (-0.0040, 0.0002)	0.08
Exposed after puberty (N = 272)	Total PBB (ppb)	0.0668 (-0.3514, 0.4851)	0.75	0.4567 (-0.0846, 0.9980)	0.09	0.3632 (-0.0932, 0.8196)	0.11
	Sex	1.2534 (-0.0733, 2.5801)	0.06	1.8295 (0.1125, 3.5465)	0.03	0.0207 (-1.4271, 1.4685)	0.97
	CD8T	31.4019 (14.9959, 47.8078)	0.0002	21.5918 (0.3594, 42.8242)	0.04	-12.2362 (-30.1395, 5.6670)	0.17
	CD4T	-12.4024 (-23.0683, -1.7364)	0.02	-27.3702 (-41.1739, -13.5664)	0.0001	-24.4398 (-36.0792, -12.8005)	4.79e-05
	B cell	7.4629 (-5.3898, 20.3155)	0.25	43.6601 (27.0264, 60.2938)	4.68e-07	-10.8411 (-24.8667, 3.1846)	0.12
	NK	0.8887 (-13.9872, 15.7647)	0.90	-2.8209 (-22.0732, 16.4314)	0.77	3.1872 (-13.0464, 19.4208)	0.69
	Mono	-6.0204 (-24.3256, 12.2848)	0.51	10.6111 (-13.0793, 34.3014)	0.37	6.3611 (-13.6147, 26.3369)	0.53
	Lipids (mg/dL)	-0.0023 (-0.0054, 0.0008)	0.14	-0.0006 (-0.0046, 0.0035)	0.78	-0.0029 (-0.0063, 0.0005)	0.09
Women (N = 381)	Total PBB (ppb)	0.0643 (-0.2185, 0.3471)	0.65	0.1650 (-0.1683, 0.4982)	0.33	0.2802 (-0.0298, 0.5902)	0.07
	CD8T	20.5487 (10.7828, 30.3145)	4.34e-05	13.0899 (1.5813, 24.5984)	0.02	-6.3396 (-17.0438, 4.3645)	0.24
	CD4T	-10.9715 (-18.2883, -3.6546)	0.003	-23.2411 (-31.8636, -14.6186)	1.99e-07	-24.5767 (-32.5965, -16.5569)	4.04e-09
	B cell	-3.6028 (-17.8814, 10.6758)	0.62	-2.2244 (-19.0510, 14.6023)	0.79	-16.3693 (-32.0198, -0.7188)	0.04
	NK	-5.7523 (-17.1718, 5.6672)	0.32	4.6740 (-8.7833, 18.1313)	0.49	-10.3013 (-22.8180, 2.2154)	0.10
	Mono	9.3291 (-5.9489, 24.6071)	0.23	23.2354 (5.2310, 41.2397)	0.01	7.7148 (-9.0310, 24.4607)	0.36
	Lipids (mg/dL)	-0.0006 (-0.0025, 0.0014)	0.56	-0.0020 (-0.0043, 0.0003)	0.09	-0.0021 (-0.0042, 0.0001)	0.05
Men (N = 277)	Total PBB (ppb)	0.4243 (0.0551, 0.7934)	0.02	0.6236 (0.1882, 1.0591)	0.005	0.3035 (-0.0932, 0.7003)	0.13
	CD8T	36.8744 (19.4129, 54.3360)	4.33e-05	23.2623 (2.6630, 43.8615)	0.02	-5.6838 (-24.4532, 13.0857)	0.55
	CD4T	-16.7029 (-27.5518, -5.8539)	0.002	-32.1249 (-44.9234, -19.3265)	1.36e-06	-23.6738 (-35.3353, -12.0122)	8.3e-05

Men (N = 277)	Total PBB (ppb)	0.4243 (0.0551, 0.7934)	0.02	0.6236 (0.1882, 1.0591)	0.005	0.3035 (-0.0932, 0.7003)	0.13
	CD8T	36.8744 (19.4129, 54.3360)	4.33e-05	23.2623 (2.6630, 43.8615)	0.02	-5.6838 (-24.4532, 13.0857)	0.55
	CD4T	-16.7029 (-27.5518, -5.8539)	0.002	-32.1249 (-44.9234, -19.3265)	1.36e-06	-23.6738 (-35.3353, -12.0122)	8.3e-05
	B cell	4.8826 (-7.3540, 17.1192)	0.43	37.9500 (23.5146, 52.3855)	4.44e-07	-4.5334 (-17.6866, 8.6198)	0.49
	NK	3.2510 (-10.6729, 17.1748)	0.64	6.0919 (-10.3339, 22.5177)	0.46	1.2100 (-13.7568, 16.1767)	0.87
	Mono	-10.3404 (-30.3557, 9.6749)	0.31	-13.6341 (-37.2460, 9.9778)	0.25	-3.8352 (-25.3497, 17.6792)	0.72
	Lipids (mg/dL)	-0.0023 (-0.0053, 0.0007)	0.12	0.0002 (-0.0033, 0.0037)	0.91	-0.0033 (-0.0065, -0.0001)	0.04
